# Polyethylene Film Surface Modification via Benzoic Acid Grafting

**DOI:** 10.3390/polym16091291

**Published:** 2024-05-05

**Authors:** Ana Luisa Grafia, Silvia Elena Barbosa

**Affiliations:** 1 Planta Piloto de Ingeniería Química, PLAPIQUI (UNS-CONICET), Bahía Blanca 8000, Argentina; agrafia@plapiqui.edu.ar; 2 Departamento de Ingeniería Química, Universidad Nacional del Sur (UNS), Bahía Blanca 8000, Argentina

**Keywords:** polyethylene surface modification, grafting, aluminum benzoate complexes, hydrophobic to hydrophilic

## Abstract

A polyethylene (PE) film surface modification method is proposed via benzoic acid (BA) alkylation grafting to improve the surface affinity to polar substances. The procedure involves sequentially spraying AlCl_3_ and BA onto the heat-softened PE surface. The occurrence of the alkylation reaction was evaluated through comparative chemical, morphological, and thermal analyses. It was demonstrated that the grafting reaction of BA onto the PE film surface took place, limited to the surface layer, while preserving the bulk properties of PE. The reaction resulted in the formation of aluminum benzoate complexes, which improved the surface affinity to polar compounds. The impact of grafting on the surface properties of PE was further assessed by comparing the behavior of PE films treated with BA and untreated PE films when painted with watercolors. The PE film grafted with BA exhibited increased affinity towards watercolors, providing strong evidence of a change in surface polarity from hydrophobic to hydrophilic. These findings indicate that the proposed methodology effectively renders the PE surface paintable, even with non-toxic water-based inks, making it suitable for applications such as packaging.

## 1. Introduction

Polyethylene (PE) stands as one of the most widely utilized thermoplastics globally due to its outstanding cost/performance/sustainability ratio. Nevertheless, despite its excellent bulk properties, the inert, non-polar, and hydrophobic PE surface imposes limitations on its applications. Properties such as printability, paintability, and adhesion, which are determined by the surface affinity with polar substances, require enhancement [1,2].

To address this demand, strategies involve changing the polymer surface through chemical or physical surface modification methods. These methods offer the advantage of preserving the material bulk properties by confining the modification to a thin surface layer. Among the most industrially used methods for PE are dielectric barrier discharges, owing to their suitability for continuous processes. Consequently, oxidized products, predominantly ozonides, hydroperoxides, and peroxides, form on the PE surface, increasing its polarity and surface free-energy. However, many of these methods suffer from instability, lack of control, nonspecificity, surface degradation, susceptibility to polar liquids, heat, or friction, and diminish over time due to aging effects, which constitute their main limitation [1,2,3].

An alternative, versatile, and tailored modification method involves surface grafting of specific molecules via wet or melting processing [1,2]. These methods entail introducing a new functional group into a polymer to modify its properties or serve as an anchor point for other molecules. By employing these approaches, stable reaction products with robust anchoring bonds to the polymer can be generated. Grafting voluminous molecules, such as chains or aromatics ones, hinders their diffusion and then probably mitigates the aging effects. 

Given PE’s inherent inertness, different specific molecules, mainly organic acids and vinyl compounds, have been selected as grafting compounds [4]. For instance, Zhu et al. [5] grafted caffeic acid functionalized with ε-polylysine onto PE via reactive co-extrusion to yield antimicrobial and antioxidant active films. El-Wakil et al. [6] grafted acrylic acid onto low-density PE through melt blending to enhance the compatibility with a composite containing neat PE and rice bran impregnated with tea tree oil. Similarly, Versteeg et al. [7] improved the mechanical properties of recycled PE by grafting glycidyl methacrylate via reactive extrusion with supercritical carbon dioxide. Additionally, Wei et al. [8] enhanced the insulation properties of low-density PE and high-density PE blends by grafting 3-aminobenzoic acid via free radical grafting using solution grafting. All the above mention methods not only modify the surface of the PE but also change its bulk. 

A promising compound for modifying only the PE surface is benzoic acid (BA). BA possesses advantageous characteristics of polarity from its carboxylic group and aromaticity from its benzene ring. Thus, enhancing the PE film’s surface affinity for polar and aromatic substances could render it suitable as a carrier for polar active or functional agents. Given that organic pigments, the preferred color components in printing inks used in flexible polyethylene-based packaging, are aromatic and polar in nature, they should exhibit good adhesion and compatibility with surface rich in BA-derived groups [9].

Friedel–Crafts alkylation emerges as an effective grafting reaction to obtain a copolymer from an aromatic monomer and a hydrocarbon chain [10]. This reaction entails the alkylation of an aromatic ring and an alkyl halide using a strong Lewis acid catalyst such as aluminum chloride (AlCl_3_). Previous studies have demonstrated the successful grafting of a hydrocarbon chain, like PE, chemically bonded to the styrene benzene ring via aromatic electrophilic substitution [11,12,13,14]. Notably, in our prior work [13], we styrene-grafted a PE film by subsequently spraying catalysts and reagents directly onto its softened surface. This PE surface modification method could be seamlessly integrated into continuous film production as a direct and expeditious post-processing treatment. Taking into account these results, this alkylation reaction seems promising for use for BA grafting onto PE.

The current study aims to enhance PE surface affinity to polar substances through BA grafting using a direct and rapid method that preserves PE bulk properties. The modification procedure involves a spraying methodology where the reagents and catalyst are sprayed onto a heat-softened PE film surface. The occurrence of the reaction was determined through comparative chemical, morphological, and thermal analyses, employing five different and independent analytical techniques. Surface compatibility with polar compounds was verified through contact angle measurements, surface free energy calculations, testing with a dyna pen, painting with water-based paints, and testing paint adhesion.

## 2. Experimental

### 2.1. Materials

PE blown films, 80 μm thick, made from LDPE 203, were provided by Dow Polisur, Bahía Blanca, Argentina (Mw: 229,300 g/mol, Mn: 22,500 g/mol). BA (ACS) and anhydrous AlCl_3_ (≥98% purity), both obtained from Merck, Darmstadt, Germany, were used as reagents. N-heptane and absolute ethanol, both obtained from Cicarelli, Buenos Aires, Argentina, with a purity higher than 99.5%, were used as solvents. Distilled water and diiodomethane, 99% purity, from Cicarelli were used for contact angle measurements. A red watercolor (0.37 g of dry paint per water mL) with permanent red tonality, pigment: PR170 C_26_H_22_N_4_O_4_ CAS: 2786-76-7 and blue tempera (5 g paint per water mL) with ultramarine blue tonality, pigment: PB29 Na_6_Al_4_Si_6_S_4_O_20_ CAS: 57455-37-3, both from Alba (professional artistic line), were used for paintability tests. 

### 2.2. Grafting Reaction Procedure

PE film squares (15 × 15 cm) were surface-softened by heating at circa 95 °C using an infrared system. It was previously demonstrated that PE is softened but not melted at this temperature [13]. The softened PE surface was consecutively sprayed with a suspension of 155 mg of AlCl_3_ in 25 mL of n-heptane and with a solution of 500 mg of BA in 25 mL of absolute ethanol. The pH of the ethanol solution was adjusted to 3 by addition of HCl solution. The spray equipment was placed 15 cm away from the film surface. It had a conventional 120° fan nozzle connected to a dry air stream at 3 bars. Experiments were performed at room temperature with a flow rate of 6 mL/min. It is important to note that the solvent is evaporated during spraying. In order to remove residual unreacted reagents and not grafted compounds, the modified films were sonicated for 2 h in absolute ethanol and then dried for 48 h under an extractor hood. Modified sonicated films were named PE-BA.

### 2.3. Characterization

The first evidence of a reaction occurrence was assessed by determining the aromatic presence in PE-BA with a colorimetric assay. This test is based on the aromatic identification of chloroform and AlCl_3_ proposed by Shriner et al. [15]. PE and PE-BA film samples were placed in a beaker with 20 mL of chloroform and 0.2 g of AlCl_3_ under agitation for 5 min. Then, the samples were removed from the beaker and the change in its surface color was observed with the naked eye and photographed. The chemical structure of PE, pure reagents, and PE-BA were analyzed by Fourier transform infrared spectroscopy (FTIR) in a Nicolet 520-FTIR (Waltham, MA, USA) spectrometer. Spectra were performed in two modes: attenuated total reflection (ATR) using zinc selenide crystal, and a transmission one. The effect of the reaction products on the thermal decomposition of modified films was studied with thermogravimetric analysis (TGA) in a temperature range of 30–800 °C at a heating rate of 10 °C/min under a nitrogen atmosphere. Thermogravimetric (TG) curves were recorded using Discovery TGA equipment from TA Instruments, New Castle, DE, USA.

Surface morphology and elemental composition of films were assessed by scanning electron microscopy with energy disperse X-ray microanalysis (SEM-EDX) in a JEOL-35CFelectron scanning microscope equipped with a EDAX DX4 microanalyzer, all from Tokyo, Japan. Samples were previously coated with Au in a sputter coater PELCO 91000, New York, NY, USA. EDAX DX4 allows elements to be detected from B to U with a surface penetration of 1 µm.

### 2.4. Wettability and Paintability Tests

The effect of the grafting reaction on the film surface compatibility with polar compounds was tested using qualitative and quantitative wettability and paintability tests. 

Contact angle measurements were performed in static mode according to the sessile drop method using a goniometer OCA-15 (Data Physics, Filderstadt, Germany). More than 50 drops of 1 µm of water and diiodomethane were placed onto different locations of PE and PE-BA surfaces, in a 25 °C and 50% RH room. 

The measured contact angle values of PE and PE-BA were used to calculate the respective total surface energy and its dispersive and polar component. Table 1 shows the surface energy parameters of distilled water and diiodomethane. The calculation of the surface energy was carried out using the Owens and Wendt equation [16].

(1) $$\gamma_{L}\left(1+cos\emptyset \right)=2\left[{\left(\gamma_{L}^{d}\gamma_{S}^{d}\right)}^{0.5}+{\left(\gamma_{L}^{p}\gamma_{S}^{p}\right)}^{0.5}\right]$$

where θ is the contact angle; $\gamma_{L}$ is the surface tension; and $\gamma_{L}^{p}$ and $\gamma_{L}^{d}$ are the polar and dispersive components of the testing liquid. Meanwhile, $\gamma_{S}^{p}$ and $\gamma_{S}^{d}$ are the polar and dispersive components of the tested films. The sum of the polar and dispersive components is the total surface energy or surface energy of the film $\gamma_{s}$. 

Surface energies were also tested with a quick method, widely used industrially, using dyne pens. A line was written onto the surface of each film (PE and PE-BA) using dyne pens of 38 and 44 dynes/cm (Lemdisa S. A., Buenos Aires, Argentina). If the ink remains as a line without forming individual droplets, then the surface has a dyne level at least as high as the ink used. If the line is continuous, without the presence of individual drops, the surface has a dyne level at least as high as the dyne pen used.

The effect of the grafting on the paintability of the PE was comparatively and qualitatively tested by painting the surface of PE and PE-BA films with red watercolor and blue tempera. Paint coverage was observed with the naked eye and photographed for later analysis. Furthermore, only on the films painted with tempera, the paint adhesion was evaluated by a tape test according to ASTM 3359-02 [18]. The classification of the results is based on the percentage of removed area (r.a.) and divided into six categories, from 5B (none r.a.) to 0B (>65% r. a.). The r.a. was calculated using the image analysis software Image J Java 8. 

## 3. Results and Discussion

Reaction occurrence and effectiveness were evaluated with a comparative chemical, thermal, and morphological analysis of the PE and PE-BA films.

**Figure 1:**
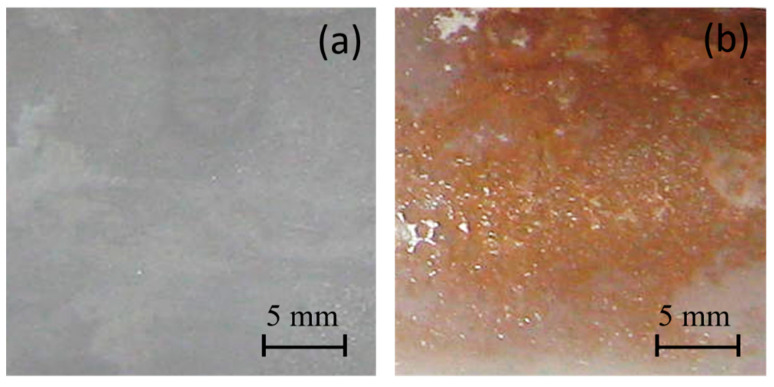
Photographs after chloroform and AlCl_3_ test of films from (**a**) PE and (**b**) PE-BA.

First, we analyzed the chemical reaction occurrence with a colorimetric assay. Figure 1 shows the surface photographs of the PE and PE-BA films after testing. The PE-BA film shows an orange appearance giving a positive test result, which evidences the aromatic presence from grafted BA; in contrast, no color changes are detected on neat PE. 

**Figure 2:**
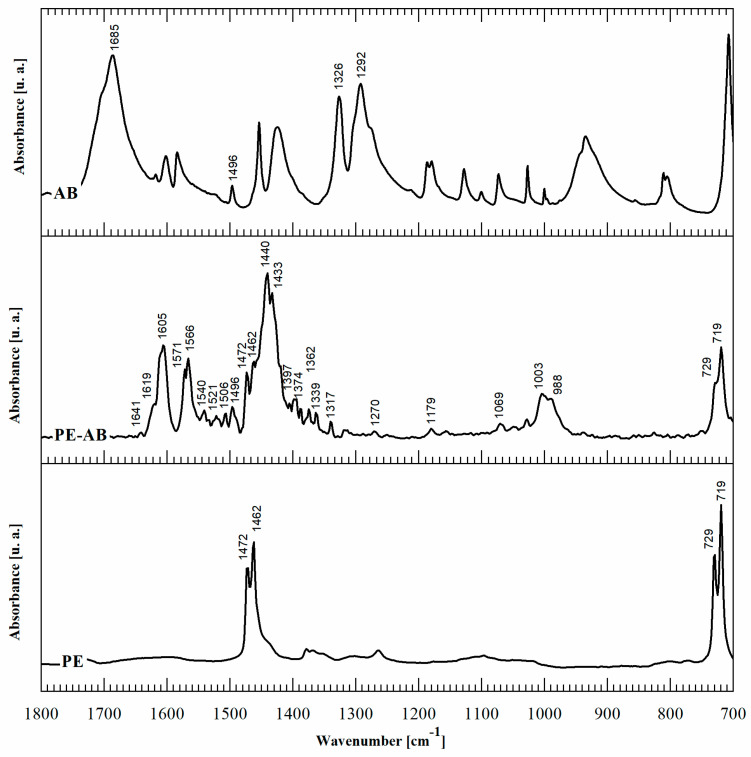
FTIR ATR spectra of BA, PE-BA, and PE.

Second, in order to elucidate the chemistry of the reaction product, FTIR-ATR spectroscopy was performed. The FTIR spectra of PE-BA compared with those of PE and BA are shown in Figure 2. The main spectrum peak assignments are summarized in Table 2. In addition to the typical PE bands [19], the spectra of PE-BA exhibit new bands attributed to the reaction product, particularly in the range of 1700–700 cm^−1^. The aromatic nature of the graft product is revealed by the band at 1496 cm^−1^ corresponding to the CC stretching vibration in the plane of the aromatic ring [20,21,22], while the rest of the BA characteristic bands undergo notable changes and shifts. One of these is the absence of the bands assigned to the carboxyl group (COOH), at 1685 cm^−1^, 1326 cm^−1^, and 1292 cm^−1^ [20,21] of BA, which allows the inference that the graft occurred through the acid group.

A detailed analysis of the new spectrum bands of PE-BA revealed that the aluminum incorporated through AlCl_3_ was part of the reaction product. The bands assigned to the carboxylate ion (COO-) associated with Al were identified. Olafsson et al. [30], Olafsson and Hildingsson [31], and Olafsson et al. [35] studied the organic acid migration in laminated packaging and its adhesion effect between sheets of PE and aluminum, by analyzing the FTIR spectra of this system. Based on these studies, the band at 1397 cm^−1^ in PE-BA can be assigned to the carboxylate ion (COO-) in contact with aluminum surfaces. The pair of bands at 1405 and 1521 cm^−1^ have also been identified as aluminum carboxylates [29]. Consistently, the bands at 1540 and 1506 cm^−1^ are attributed to benzoate ion complexed with Al [28]. Other absorption bands assigned to aluminum chemical bonds in PE-BA are as follows: the bands at 1179 and 1069 cm^−1^ are attributed to link Al-O-C [33]; the bands at 1003 and 990 cm^−1^ are specific to Al-O [30,34]; and at 1270 cm^−1^, the band is assigned to the CH_2_-Al bond [32]. These results allow us to corroborate that grafting occurs through the acidic group of BA. In turn, AlCl_3_ not only acts as a catalyst, but also participates in the formation of the reaction product. Furthermore, Al is involved in BA anchoring to the PE matrix. Al bonds with PE chains through the methylene group (CH_2_-Al) and the carboxylic group of BA. 

Moreover, AlCl_3_ typical infrared absorption bands were not detected in any of the modified film spectra. In particular, neither the characteristic band of AlCl_3_ at 1650 cm^−1^ nor the less intense band at 1158 cm^−1^ assigned to the ClO^4−^ group were identified [36,37]. The absorption bands in the modified spectra films assigned to aluminum bindings were attributed to the reaction product. Furthermore, no Cl infrared absorption bands were identified on modified film spectra; this element was released during the reaction experiment, not forming part of the product [38]. This was expected because AlCl_3_, as demonstrated above, acts both as a catalyst and reagent and the surplus is dissolved in ethanol [39], the solvent used for the film-washing process.

It is important to note that the PE-BA spectrum includes several bands that can be assigned to aluminum aromatic complexes. In the literature, it was demonstrated that aluminum with BA forms different coordination complexes of aluminum benzoates [27,40]. In particular, AlCl_3_ was previously used as an Al source to obtain different aluminum carboxylate complexes, as in the present study. Pękal and Pyrzynska [41] determined the flavonoid content through the aluminum-flavonoid complexes formed from the reaction of flavonoid, an aromatic compound with carbonyl groups, with a solution of AlCl_3_. Yokel et al. [42] prepared Al citrate from AlCl_3_·6H_2_O and citric acid trisodium salt dehydrate. Karlik et al. [43] studied the interactions between Al(III) and carboxylate ligands from salts of lactate, citrate, and EDTA prepared by mixing AlCl_3_ and the corresponding sodium salt. Motekaitis and Martell [44] studied the complexation of Al(III) with hydroxyl carboxylic acids using potentiometric methods. They used AlCl_3_·6H_2_O and crystalline monopotassium salts of citric acid, tartaric acid, gluconic acid, saccharic acid, glyceric acid, bis(hydroxyethyl) glycine, and catechol as reagents.

Table 3 summarizes the benzoate complex configurations depending on the amount and type of associations generated between the metal and the carboxylate group (COO-) of the acid: mononuclear monodentates and mono- or binuclear bidentates. The absorption spectra corresponding to these complexes have been analyzed in detail in reports focused on the absorption of BA in minerals containing aluminum [25,45,46], aluminum hydroxides [23,24,40], and alumina [47]. In the PE-BA spectrum (Figure 2, Table 2), the bands at 1619, 1641, 1571, 1440, and 1317 cm^−1^ are attributed to monodentate complexes [23,24,25,26]. The bands at 1433, 1605, and 1566 cm^−1^ in the PE-BA spectrum are attributed to bidentate complexes, chelates, or bridge [23,27]. A higher proportion of bands assigned to the monodentate complexes is observed, suggesting a reaction product with a predominance of this complex type.

**Figure 3:**
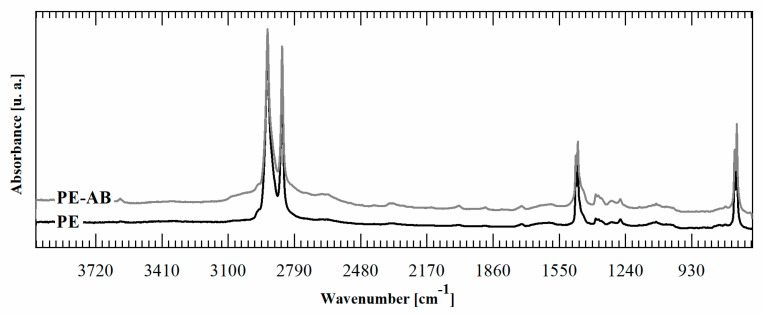
FTIR transmission spectra of PE-BA and PE.

It is important to note that the reaction occurs only in the film surface. To prove this point, an FTIR was performed in transmission mode on PE-BA and PE films. Contrary to the FTIR-ATR spectra, in transmission spectra it is not possible to detect bands corresponding to the graft reaction products, as is shown in Figure 3. ATR-FTIR spectroscopy is a surface technique with a penetration depth that depends on IR wavelength, the refractive index of the sample, the refractive index of used crystal, and angle of incident light. The depth of penetration is up to 2 µm when organic materials are tested in a standard setup ATR-FTIR accessories using zinc selenide crystal and a 45° incident angle [48]. Then, considering that the PE film used as a substrate for the reaction is 100 microns thick, the reaction is circumscribed to its surface zone, penetrating less than 2% of the whole film thickness; then, the major volume of the film remains unaltered.

**Figure 4:**
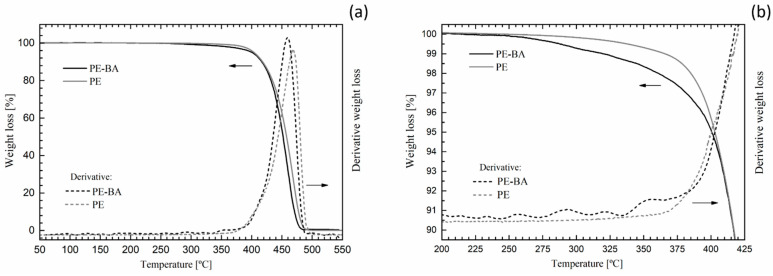
PE and PE-BA thermogravimetric curves: complete (**a**) and expanded area (**b**).

Third, we compared the thermal decomposition behavior of PE-BA with that of pure PE. Figure 4 shows complete (Figure 4a) and expanded (Figure 4b) thermogravimetric curves of PE and PE-BA. Both weight loss and the derivative of weight loss are depicted in order to assess thermal events related to reaction products resulting from surface modification. Both thermograms exhibited an overall similar behavior (Figure 4a), typical of PE, with a significant drop at around 450–460 °C associated with hydrocarbon degradation [49,50]. However, the thermogram of PE-BA shows a slight shift towards higher temperatures compared to that of PE. This shift is clearer in the derivative curves. This is the first thermal evidence of the presence of reaction products on PE-BA because aluminum compounds act as PE stabilizers, causing the loss weight at higher temperature values [51,52].

The main differences in the thermal behavior of PE and PE-BA were detected in the temperature range from 200 °C to 450 °C (Figure 4b). PE-BA begins to lose weight at around 225 °C, earlier than PE, which starts at around 290 °C. At 350 °C, PE-BA has lost 1% more mass than in PE. In the derivative curve of PE-BA, several small peaks associated with weight loss between 250 °C and 380 °C can be identified, with the largest one around 350 °C. This is attributed to the degradation of aluminum benzoate complexes formed during the grafting reaction, as identified with FTIR analysis. Furthermore, thermograms of this type of complex formed on clay surfaces [46] and of Ni-Al-benzoate obtained for amoxicillin absorbent [53] have shown degradation steps in the range of 200 °C to 400 °C, consistent with the assessed temperature range. Note that the degradation temperatures of AlCl_3_ and BA are lower than the events detected in PE-BA [54]; thus, the presence of these reagents was not detected, as expected.

**Figure 5:**
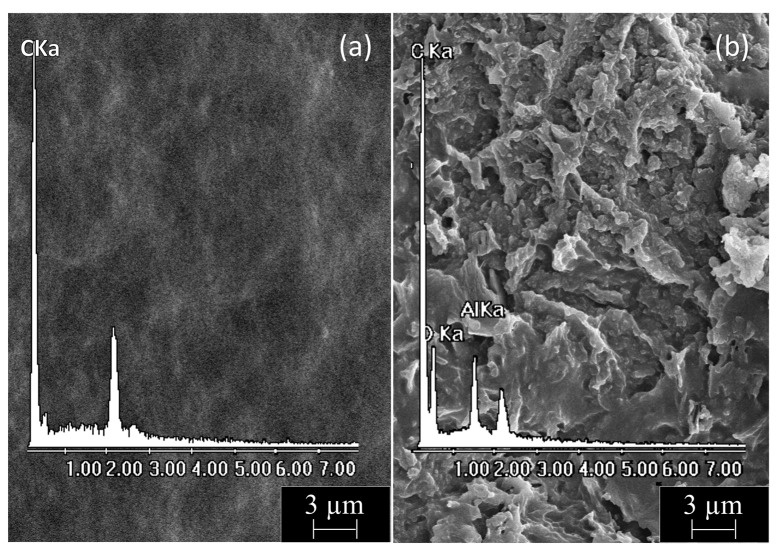
SEM micrograph (5000×) with corresponding EDX spectrum from film surfaces of (**a**) PE and (**b**) PE-BA.

Finally, we performed a comparative and combined morphological–elemental analysis of PE-BA and pure PE surfaces. SEM micrographs of both film surfaces with their corresponding EDX spectra are shown in Figure 5. PE films (Figure 5a) displayed a smooth homogeneous surface containing only C according to the chemical composition of this polymer. In contrast, PE-BA films (Figure 5b) exhibited a wrinkled and irregular surface containing C, O, and Al, according to the appearance of a growing specimen in the form of a surface layer and with the chemical composition of the previously proposed aluminum benzoate complexes. It should be noted that Cl was not distinguished during analysis [54], so it was ruled out that it was part of the graft, as predicted for the reaction and as suggested by the FTIR analysis.

**Figure 6:**
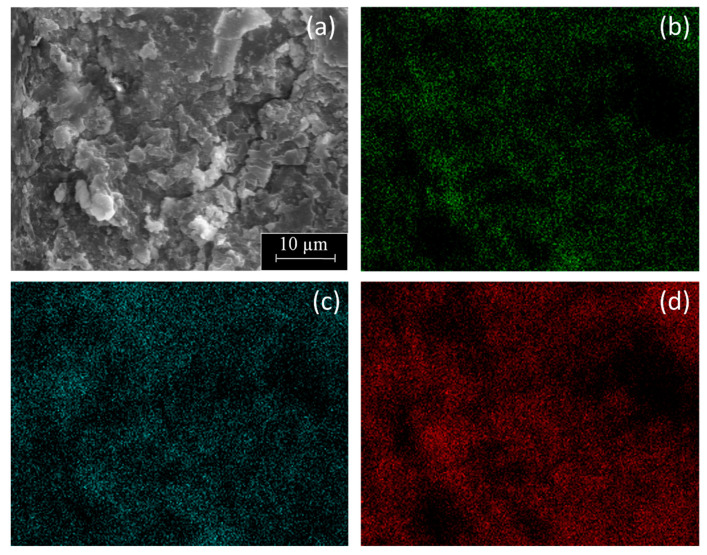
SEM micrograph (2000×) of PE-BA film surface (**a**) with corresponding elemental EDX mapping of (**b**) O, (**c**) Al, and (**d**) C.

Figure 6 includes a SEM micrograph with corresponding EDX mapping of C, O, and Al of the PE-BA surface. It was shown that both C, main component of PE, and O, as well as Al, components of the graft, were uniformly distributed throughout the analyzed surface. Therefore, this result revealed that the reaction on PE surface was uniform and massive. In fact, the PE-BA surface was differentiable from the PE surface even at 100×, the smallest magnification tested by SEM. Furthermore, from visual examination, PE-BA and PE films did not seem to be exactly equal. Even the texture to the touch of both films differed, that of PE-BA being rougher. These observations demonstrated a good reaction extent covering the complete, treated PE film surface.

In order to validate that the grafting reaction changes the surface polarity of PE films, the wettability was evaluated using contact angle measurements and surface energy calculations. Table 4 shows the measured values of the contact angle using water and diiodomethane and the calculated values of the dispersive ($\gamma_{s}^{d}$) and polar ($\gamma_{s}^{p}$) components and the total ($\gamma_{s}$) of the surface energy for PE-BA and PE. The water contact angle for PE is 5° greater than 90°, meaning that its surface is strongly hydrophobic, as is expected. The measured angles are comparable to those reported in the literature, ranging from 90 to 100° for PE films [55,56,57,58,59]. In contrast, the value of the water contact angle for PE-BA is more than 10° lower than that of PE, suggesting a hydrophilic nature as it falls below 90°. This clearly indicates the transformation of the surface character of PE from hydrophobic to hydrophilic upon grafting reaction. Furthermore, compared to aromatic polymers such as polystyrene, which typically exhibit a water contact angle of around 94° [60,61], the water contact angle of PE-BA is even lower, indicating a more polar surface. PE-BA water contact angle values are similar to an oxidized polystyrene (PS) with an oxygen atom percentage slightly higher than 3%, that is equivalent to a 0.013 oxygen atom/Å^2^ [60]. These results are in concordance with the chemistry structure of the grafting products suggested by FTIR analysis. Benzoate complexes linked to PE by bridges between the carboxylic group and Al, but also free carboxylic acid groups, were identified, which together give greater polarity compared to the grafting of neutral aromatic groups. Meanwhile, the values of the contact angle with diiodomethane did not change significantly. Diiodomethane is a slightly polar liquid with a surface energy less than half that of water, but sufficient to produce easily measurable contact angles. This characteristic makes it ideal for complementing water contact angle measurements in surface energy analysis. However, due to its slight polarity and primarily dispersive forces, it is less sensitive to changes in the substrate polarity [62]. The measured values are comparable to those reported in the literature for untreated PE, which are 51° [63], 55.6° [56], and 52.8° [57], among other similar values.

Surface energy parameters, calculated from contact angle measurements using Equation (1), show that the values of $\gamma_{s}$ and $\gamma_{s}^{p}$ for PE-BA are higher than for PE. The calculated values for PE agree with the literature for LDPE films, which reports values around 30 mJ. m^−2^ [55,56,57,59]. Although the values of total surface energy are quite close between them, the values corresponding to PE-BA remained slightly higher, denoting the change in the total polarity of the surface. Meanwhile, the greatest changes are observed in the values of the polar component of PE-BA, which increases more than 4 times compared to PE. The percentage of the polar component, with respect to the total surface energy, increased from 3% to 11%. This change can be attributed to a polarity increase by the surface grafting products. Similar delta and total surface energy values were reported by Rytlewski and Żenkiewicz, [64] for PS and PS oxidized by laser treatment, using a pulse number 10, with an atomic oxygen concentration close to 6.4%. This oxygen concentration is at least 2 times higher than the value informed by the work of Bekele and Tsige [60], discussed previously for similar water contact angle of an oxidized PS. 

**Figure 7:**
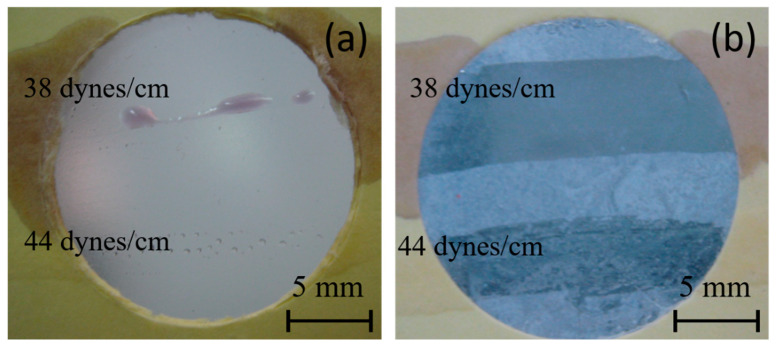
Photographs of dyne pen testing, using 38 and 44 dynes/cm pens, on the surfaces of (**a**) PE and (**b**) PE-BA.

A commonly used semiquantitative analysis to determine surface energy in the industry is the dyne pen test. In Figure 7, the results of this test for both PE and PE-BA samples are presented. No continuous lines are observed on the PE surface; only drops are visible with the 38 dynes/cm pen, and even smaller individual drops with the 44 dynes/cm pen. Conversely, continuous lines are observed on the PE-BA samples drawn with both the 38 and 44 dynes/cm pens. These results suggest that the surface energy of PE is below 38 dynes/cm, while that of PE-BA is even higher than 44 dynes/cm. While the behavior of PE aligns with expectations, that of PE-BA surpasses what was calculated from the contact angle (refer to Table 4). These disparities may stem from the fact that the contact angle is influenced not only by surface chemistry but also by surface topography. As shown in SEM images, PE-BA is rougher than PE.

Generally, industrial printing requirements are approximately 38–40 dynes/cm for solvent-based inks and around 42–22 dynes/cm for water-based inks [9]. Accordingly, PE-BA films would be suitable for painting with water-based inks. In order to demonstrate this fact, several experiments using watercolor and tempera paints were performed on both films.

**Figure 8:**
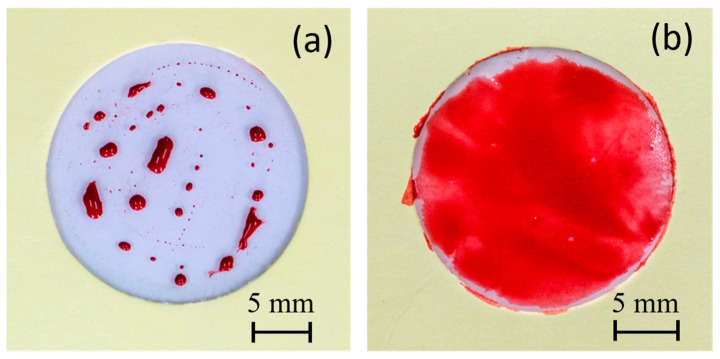
Photographs of painting experiments with red watercolor on the surfaces of (**a**) PE and (**b**) PE-BA.

**Figure 9:**
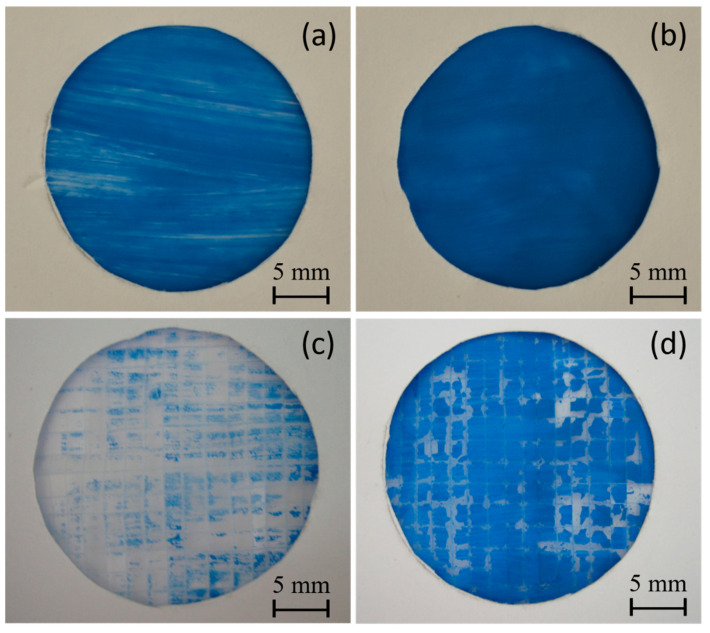
Photographs of painting experiments with blue tempera on the surfaces of (**a**) PE and (**b**) PE-BA and after adhesion test (**c**) PE and (**d**) PE-BA.

Two different water-based paints, red watercolor and blue tempera, were utilized to conduct a comparative paintability test on PE and PE-BA films. In Figure 8, the surfaces painted with red watercolor are depicted for both PE (a) and PE-BA (b) films. While the watercolor evenly covers the entire surface of the PE-BA film, it is repelled from the PE surface, forming independent droplets. The increased hydrophilicity of PE-BA is evident, confirming the alteration in surface activity resulting from the chemical bonding of polar and aromatic groups onto the PE surface. Figure 9 displays the surfaces painted with blue tempera for both PE and PE-BA. Although both surfaces are painted, the coverage is notably better on PE-BA. Brush strokes fail to completely cover the PE background, leaving white spaces, a phenomenon absent on PE-BA. This change in behavior is more pronounced when using watercolor, as it has a higher water proportion and lower viscosity compared to tempera. Additionally, the watercolor pigment, naphthol red (C_26_H_22_N_4_O_4_), contains aromatic rings in its structure, rendering it more compatible with other aromatic compounds such as benzoates present on the PE-BA surface.

Contact angle measurements, dyne pen testing and painting with watercolor were also performed with PE-BA samples that were stored for more than nine months. No changes in the performance of such samples compared with new ones were detected. This behavior could be attributed to the thermodynamic stability of the benzozed aluminum complexes generated on the PE-BA surface and to their voluminous molecules, since, in addition, the complexes tend to associate with each other, which makes their diffusion through the PE matrix difficult [65]. According to these results, the aging effect of grafting could be not considered as an important detrimental factor of the surface properties. 

In Figure 9, photographs c and d display the surfaces of the PE and PE-BA films, respectively, after undergoing an adhesion test [18]. The improvement in paint adhesion is evident, as the surface of the PE film is nearly devoid of paint, while the PE-BA surface retains good coverage. The estimated values of r.a. for PE are approximately 91%, whereas for PE-BA, they are around 17%, representing an adhesion enhancement of more than 5 times. According to the classification of ASTM 3359-02, these results correspond to 2B (15–35% r.a.) for PE-BA and 0B (>65% r.a.) for PE. Similar findings for neat PE have been previously reported by Ataeefard [66] and Grafia et al. [13].

These results demonstrate that the surface modification effectively increases the affinity of polar substances with the PE surface, owing to the grafting of oxygenated and aromatic groups. In particular, benzoates and derivatives enhance polyester dyeing by serving as dye carriers [67,68]. Polystyrenes recovered from waste are converted into dye absorbents by grafting 1,2,4-benzenetricarboxylic anhydride [4] or maleic anhydride [69] through Friedel–Crafts reactions, employing AlCl_3_ as a catalyst. These graft molecules, which share chemical structures in common with those generated on PE-BA, increase the dye-receptive sites of the polymer. The dye–polymer interactions proposed in these works, such as electrostatic interaction, hydrogen bonding, n-π interaction, π-π interaction, and complexation, could be similar to those occurring with PE-BA. All of these interactions create different dye-anchoring pathways that enhance the polymer–dye affinity. In contrast, interactions of dyes with oxidized polymers, such as through corona treatment, primarily occur with the hydroxyl, carbonyl, and other oxygen-containing groups on their polarized surface [70].

## 4. Conclusions

A method for introducing hydrophilic characteristics to PE film through grafting reactions is proposed. This method involves an alkylation reaction on the molten film surface using AlCl_3_ as a catalyst, enabling the grafting of BA. Surface reaction occurrence and identification of reaction products were assessed through a comparative chemical, thermal, and morphological analysis of pure PE and reacted films, previously extracted with ethanol.

The presence of aromatic compounds on the modified film surface was initially confirmed using the chloroform and AlCl_3_ aromatic identification test. The chemical nature of the reaction product was determined through FTIR-ATR spectroscopy analysis, revealing that BA is indeed grafted to PE and that aluminum is part of the reaction product, resulting in a mixture of mono and bidentate benzoate complexes. The existence of such complexes on PE-BA was further confirmed by thermogravimetric analysis. Additionally, it was demonstrated that the chlorine incorporated through AlCl_3_ is not present in the modified films. Furthermore, morphological-elementary analysis provided additional evidence of reaction occurrence.

It was also demonstrated that the grafting reaction of BA onto PE occurs exclusively on the film surface, transforming PE films from hydrophobic to hydrophilic and subsequently improving surface affinity towards polar compounds. This change in surface polarity was assessed using diverse qualitative and quantitative methods: contact angle measurements, surface free energy calculations, dyne pen test, paintability with watercolors and tempera, and adhesion tests for these water-based paints. These results can be explained by a synergistic effect of included polarity and aromaticity PE surface from BA-grafted molecules. Thus, it not only increases compatibility with polar compounds but also with aromatic compounds. Considering that many inks and pigments are aromatic in nature, this is an added advantage. Furthermore, no aging effects were detected.

This method could be scalable for direct application in blown film processes. It can be applied immediately above the freeze line, using two successive spraying annular systems. At this location of the PE bubble, the surface film is molten, allowing reagents to be sprayed successively and reacts at the rates of the process. Furthermore, the modification proposed in this work could be extended to other thermoplastics polymeric substrates such as polypropylene and with other grafting molecules such as salicylic acid. In this way, the surface of the polymers could be tailored according to each specific application required.

## Figures and Tables

**Table 1:** Surface tensions ($\gamma_{L}$) of testing liquids and their dispersive ($\gamma_{L}^{d}$) and polar ($\gamma_{L}^{p}$) components [17].

Liquid	$\gamma_{L}^{d}$ [mJ. m^−2^]	$\gamma_{L}^{p}$ [mJ. m^−2^]	$\gamma_{L}$ [mJ. m^−2^]
water	21.8	51.0	72.8
diiodomethane	50.42	0.38	35.00

**Table 2:** FTIR identification and assignment of the main absorption bands for PE-BA, PE, and BA *.

Frequency [cm^−1^]			Assignment	References
PE-BA	PE	BA		
		1685	C=O free carboxylic acid stretching mode	[20,21]
**1641**			C=O stretching vibration, in monodentate aluminum benzoate complexes (1645 cm^−1^).	[23]
**1619** **1571** **1440** **1317**			Vibrations related to monodentate aluminum benzoates complexes (1622 cm^−1^, 1571 cm^−1^, 1440 cm^−1^, 1317 cm^−1^)	[24,25,26]
**1605** **1566** **1433**			C-O in bridge or bidentate complexes	[23,27]
**1506** **1540**			-COO^−^ benzoate ion (1550 cm^−1^) and oriented benzoate on aluminum oxide (1500 cm^−1^)	[28]
**1521** **1405**			-COO^−^Al^+^ aluminum carboxylate (1523–1410 cm^−1^)	[29]
**1496**		1496	C-C of aromatic ring	[20,21,22]
1472 1462	1472 1462		CH bending deformation	[19]
**1397**			-COO^−^ carboxylate ion on aluminum surface	[30,31]
1378	1378		CH_3_ symmetric deformation	[19]
1306	1306		CH_3_ twisting deformation	[19]
		1326 1292	C-O of COOH-coupled stretching vibrations	[21]
**1270**			CH_2_-Al	[32]
**1179** **1069**			Al-O-C	[33]
**1003** **990**			Al-O	[30,34]
721 719	721 719		CH_2_ rocking deformation	[19]

* The bands assigned to the grafting product are highlighted in bold.

**Table 3:** Aluminum benzoate coordination complexes [24].

Salt	Monodentate Complexes	Bidentate Complexes	
		Mononuclear	Binuclear
		Chelate	Bridge
Aluminum benzoates	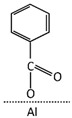	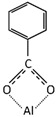	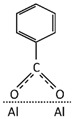

**Table 4:** Water and diiodomethane contact angle and surface energy of PE-BA and PE.

Sample	Water Contact Angle [°]	Diiodomethane Contact Angle [°]	Surface Energy [mJ. m^−2^]		
			$\gamma_{s}^{d}$	$\gamma_{s}^{p}$	$\gamma_{s}$
PE	95 ± 2	52 ± 1	31.71	0.95	32.71
PE-BA	84 ± 3	51 ± 2	30.97	4.04	35.00

## Data Availability

The original contributions presented in the study are included in the article, further inquiries can be directed to the corresponding authors.
